# FOXA2 activates HIF2α expression to promote tumor progression and is regulated by the E3 ubiquitin ligase VHL in renal cell carcinoma

**DOI:** 10.1016/j.jbc.2023.105535

**Published:** 2023-12-10

**Authors:** Dongjun Yang, Qixiang Li, Peifen Lu, Dongliang Wu, Wenyang Li, Xingjun Meng, Mengying Xing, Wenbing Shangguan, Bing Chen, Jie Yang, Zhihong Zhang, Zengjun Wang, David C.S. Huang, Quan Zhao

**Affiliations:** 1 The State Key Laboratory of Pharmaceutical Biotechnology, Department of Hematology, The Affiliated Drum Tower Hospital of Nanjing University Medical School, China-Australia Institute of Translational Medicine, School of Life Sciences, Nanjing University, Nanjing, China; 2 Department of Urology and Pathology, The First Affiliated Hospital of Nanjing Medical University, Nanjing, China; 3 Department of Medical Biology, The Walter and Eliza Hall Institute of Medical Research, University of Melbourne, Melbourne, Victoria, Australia

**Keywords:** FOXA2, renal cell carcinoma, HIF2α, VHL, gene expression, cell proliferation

## Abstract

Renal cell carcinoma (RCC) is a frequent malignancy of the urinary system with high mortality and morbidity. However, the molecular mechanisms underlying RCC progression are still largely unknown. In this study, we identified FOXA2, a pioneer transcription factor, as a driver oncogene for RCC. We show that FOXA2 was commonly upregulated in human RCC samples and promoted RCC proliferation, as evidenced by assays of cell viability, colony formation, migratory and invasive capabilities, and stemness properties. Mechanistically, we found that FOXA2 promoted RCC cell proliferation by transcriptionally activating HIF2α expression *in vitro* and *in vivo*. Furthermore, we found that FOXA2 could interact with VHL (von Hippel‒Lindau), which ubiquitinated FOXA2 and controlled its protein stability in RCC cells. We showed that mutation of lysine at position 264 to arginine in FOXA2 could mostly abrogate its ubiquitination, augment its activation effect on HIF2α expression, and promote RCC proliferation *in vitro* and RCC progression *in vivo*. Importantly, elevated expression of FOXA2 in patients with RCC positively correlated with the expression of HIF2α and was associated with shorter overall and disease-free survival. Together, these findings reveal a novel role of FOXA2 in RCC development and provide insights into the underlying molecular mechanisms of FOXA2-driven pathological processes in RCC.

Renal cell carcinoma (RCC) is among the top 10 malignant neoplasms in men worldwide and encompasses a heterogeneous group of histological subtypes, including the most commonly diagnosed clear cell renal cell carcinoma (ccRCC) (1, 2). In addition to nephrectomy, targeted therapies and immunotherapy have been developed to treat RCC (3). Nevertheless, the response is varied and poor, and most patients eventually develop metastases and drug resistance, with the median survival of patients remaining less than 3 years (3).

Genetically, people predisposed to RCC largely harbor defective von Hippel‒Lindau (VHL), a tumor suppressor gene encoding a type of E3 ubiquitin ligase (4). VHL inactivation usually initiates both hereditary (VHL Disease) and sporadic ccRCCs through deregulation of HIF2 (5). Activation of the HIF2 pathway leads to the transcription of genes, such as VEGF and PDGF-β, which promote angiogenesis and tumorigenesis (4, 5). However, studies have revealed that VHL inactivation is not sufficient for RCC malignant transformation (6). Therefore, the accumulation of other genetic and epigenetic changes might be required (7). Thus, a molecular understanding of the pathogenic mechanisms of RCC progression and the development of new therapeutic targets for RCC are of great importance.

FOXA2, also known as hepatocyte nuclear factor 3-β (HNF3B) (8), is a pioneer transcription factor that belongs to the forkhead (FOX) family (9) and plays essential roles in cellular processes such as metabolism, organogenesis, immunity, and gene regulation (10, 11, 12, 13). FOXA2 is required for the formation of the node and notochord, and its absence results in embryonic lethality (13). FOXA2 can bind to its consensus DNA-binding site (TAAACA) at the gene promoter region and regulate gene expression (14). Studies have shown that FOXA2 could function as a tumor suppressor by selectively binding to the promoter of several genes related to epithelial–mesenchymal transition (EMT), such as Slug, CDH1 or Zeb2, to suppress tumor migration and progression in lung cancer, breast cancer or liver cancer (15, 16, 17). Conversely, FOXA2 has also been reported as an oncogene to facilitate the expression of genes such as HES6, SOX9, or MUC2 in neuroendocrine prostate cancer or Barrett’s metaplasia (18, 19). With its forkhead domain structure highly resembling that of linker histone H1 (20), FOXA2 can bind to nucleosomes directly to facilitate an open chromatin configuration, enabling the recruitment of other transcription factors and regulating gene transcription (21). High expression of FOXA2 has been shown in neuroendocrine lung cancer and triple-negative/basal-like breast carcinoma (22, 23). However, the role of FOXA2 in renal cell carcinoma progression remains unknown.

In this study, we identified FOXA2 as a key driver oncogene that activates HIF2α expression by directly binding to the EPAS1 (or HIF2A, encoding HIF2α) promoter, promoting RCC cell proliferation *in vitro* and *in vivo*. Moreover, we found that FOXA2 could be targeted by the E3-ubiquitin ligase VHL on K264, which regulates protein stability. Thus, our study reveals the key role of FOXA2 upstream of HIF2α during RCC progression and provides new insights for targeted therapy for patients with RCC.

## Results

### Upregulation of FOXA2 correlates with poor prognosis in RCC

**Figure 1:**
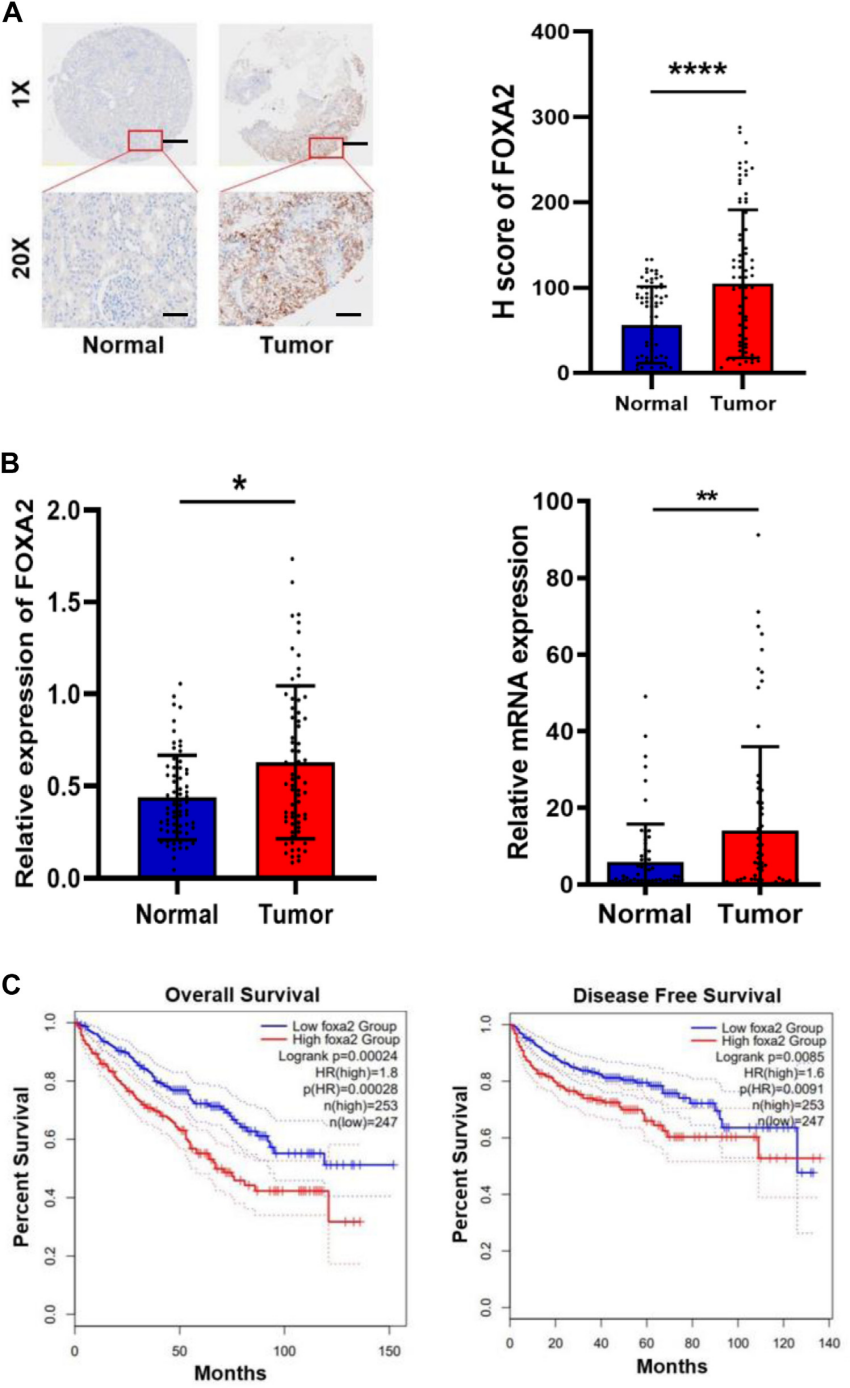
**Upregulation of FOXA2 correlates with poor prognosis in renal cell carcinoma.***A*, *left panel*: Representative images of IHC staining of FOXA2 protein in RCC tissues and matched normal tissues (n = 75). Micrographs are shown at original magnifications (x1 or x20) as indicated. Scale bars, 50 μm or 1 mm. *Right panel*: Total IHC score of FOXA2 in RCC tissues and matched normal tissues. ∗∗∗∗*p* < 0.0001. *B*, *left panel*: FOXA2 protein expression levels by quantitative analysis of the density of protein bands from Western blot in Fig. S2 relative to the normal tissues (n = 67). ∗*p* < 0.05. *Right panel*: FOXA2 mRNA levels by quantitative RT-PCR from samples in Fig. S2 relative to the normal tissues (n = 67). ∗∗*p* < 0.01. C, Kaplan‒Meier plots of overall survival and disease-free survival of 500 patients with ccRCC stratified by FOXA2 expression.

To investigate the clinical relevance of FOXA2 expression in renal cell carcinoma, we examined FOXA2 expression in renal tissue microarray (TMA) specimens from a cohort of 75 patients with RCC by immunohistochemistry using a specific anti-FOXA2 antibody (Fig. S1*A*). Immunohistochemical analysis showed significantly higher expression levels of FOXA2 in renal tumor tissues than in matched adjacent normal tissues from patients with RCC (Fig. 1*A*). In addition, we found that the expression level of FOXA2 in RCC positively correlated with the tumor stage and differentiation state of the cancer cells, although differences in lymph node metastasis and distant metastasis were not significant (Table 1). Western blot analysis and quantitative RT‒PCR confirmed that the expression levels of FOXA2 in fresh tumor tissues from 67 patients were significantly higher than those in matched adjacent normal tissues (Figs. 1*B* and S2). These results were consistent with FOXA2 expression data in RCC tissues using TCGA and GTEx sequencing datasets from the Gene Expression Profiling Interactive Analysis (GEPIA) database (Fig. S1*B*). Importantly, RCC patients with high FOXA2 expression had shorter overall survival and disease-free survival (Fig. 1*C*). Together, these results indicate that FOXA2 protein and mRNA levels were upregulated in RCC tissues and suggest that high levels of FOXA2 expression may correlate with poor prognosis in RCC.

**Table 1:** Clinicopathologic characteristics of FOXA2 expression In clear cell renal cell carcinoma patients

Characteristics	Cases	H Score of FOXA2 (mean ± SD)	*P* value^a^
	75		
Tumor size			0.6751
≥5 cm	55	101.85 ± 85.34	
≤5 cm	20	111.45 ± 92.69	
Tumor stage^b^			0.0313
I-II	52	116.44 ± 87.97	
III-IV	23	72.35 ± 58.37	
Lymph node status^b^			0.8338
N0	72	104.85 ± 88.54	
N1-2	3	94.00 ± 21.63	
Distant metastasis^b^			0.8265
M0	73	104.78 ± 87.32	
M1	2	91.00 ± 26.70	
Histological differentiation			0.0241
I-II	52	119.35 ± 92.11	
III-IV	23	70.65 ± 62.99	

a *p* values were derived using Student’s *t* test to compare values for the two parameters in each category.

b The tumor stage, lymph node status and distant metastasis were classified according to the American Joint Committee on Cancer (AJCC) TNM system.

### FOXA2 promotes cell proliferation in RCC cells

**Figure 2:**
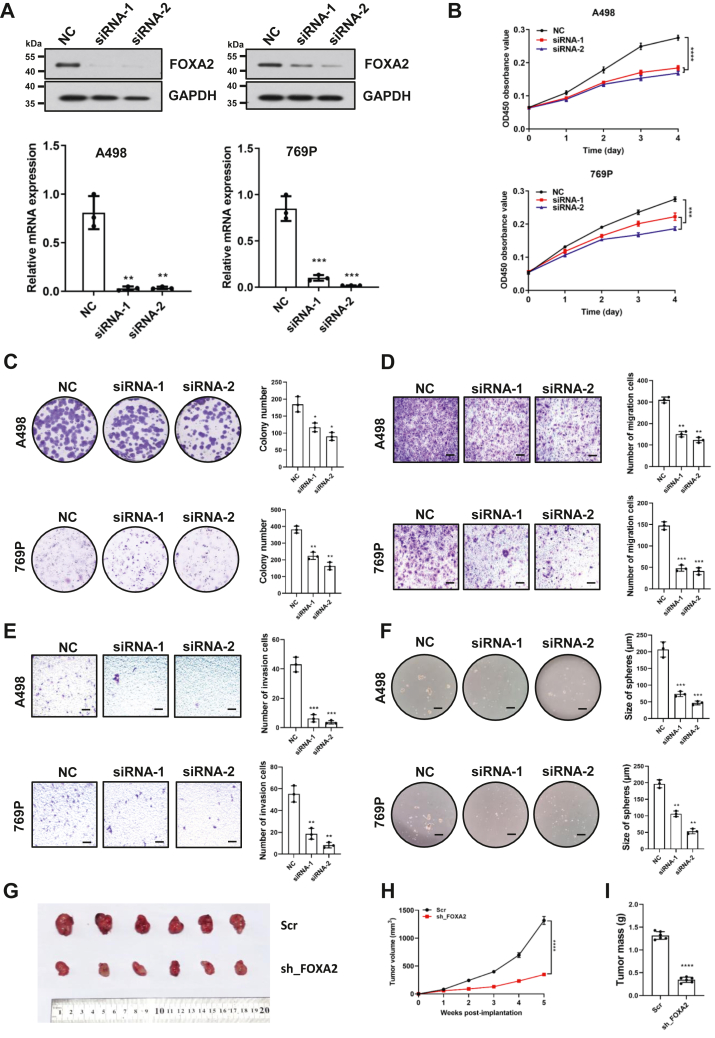
**FOXA2 promotes cell proliferation in RCC cells.***A*, Western blot and quantitative real-time PCR analysis of FOXA2 knockdown by siRNA or negative control siRNA (NC) in A498 and 769P cells. GAPDH was used as a loading control. The results are shown as the mean ± SD from three independent experiments. ∗∗*p* < 0.01. ∗∗∗*p* < 0.001. *B*, cell proliferation was determined by CCK-8 assays in A498 and 769P cells transfected with FOXA2 siRNAs or NC. The results are shown as the mean ± SD from three independent experiments. ∗∗∗∗*p* < 0.0001, ∗∗∗*p* < 0.001. *C*, colony formation assay of A498 and 769P cells with knockdown of FOXA2 or NC. The number of colonies formed by the indicated cells was quantified. The results are shown as the mean ± SD from three independent experiments. ∗*p* < 0.05, ∗∗*p* < 0.01. *D*, Transwell assay of A498 and 769P cells with knockdown of FOXA2 or NC. Scale bars, 100 μm. The number of migrated cells was quantified. The results are shown as the mean ± SD from three independent experiments. ∗∗*p* < 0.01, ∗∗∗*p* < 0.001. *E*, invasion assay of A498 and 769P cells with knockdown of FOXA2 or NC. Scale bars, 100 μm. The number of invasive cells was quantified. The results are shown as the mean ± SD from three independent experiments. ∗∗*p* < 0.01, ∗∗∗*p* < 0.001. *F*, tumor sphere formation assay of A498 and 769P cells with knockdown of FOXA2 or NC. Scale bars, 200 μm. The size of the spheres formed by the indicated cells was quantified. The results are shown as the mean ± SD from three independent experiments. ∗∗*p* < 0.01, ∗∗∗*p* < 0.001. *G*–*I*, Photograph (*G*), tumor volumes (*H*) and tumor masses (*I*) of Scr- or sh_FOXA2-treated A498 cell-derived xenograft tumors at day 35. The results are shown as the mean ± SD (n = 6). ∗∗∗*p* < 0.001. ∗∗∗∗*p* < 0.0001.

To investigate the role of FOXA2 in RCC development, we knocked down FOXA2 in two RCC cancer lines, A498 and 769P, by two independent interference RNAs, which reduced FOXA2 expression to less than 80% of the scrambled negative controls (NC) (Fig. 2*A*). Knockdown of FOXA2 significantly slowed the cell growth rate in both lines compared to NCs (Fig. 2*B*). In addition, the knockdown of FOXA2 significantly reduced the numbers of RCC cell colonies formed after culture and decreased the migratory and invasive capability of RCC cells compared to NC controls, as demonstrated by colony formation assay and transwell assay (Fig. 2, *C*–*E*). Furthermore, we observed that tumorsphere-formation activity was significantly reduced in FOXA2 knockdown cells compared to NC control cells (Fig. 2*F*). To support the role of FOXA2 in promoting RCC cell proliferation and stemness, we generated RCC patient-derived organoids using three-dimensional (3D) culture systems and transfected these organoids with shRNA to knockdown FOXA2 expression. We found that the average size and growth rate of organoids with FOXA2 knockdown were significantly smaller and slower than those seen with the scramble control (Fig. S3, *A*–*C*). Moreover, we found that RCC tumor organoids were also significantly larger in size than adjacent normal tissue organoids (Fig. S3, *D* and *E*). These results indicate that FOXA2 promotes RCC cell proliferation and maintains cancer cell stemness *in vitro*.

To test whether FOXA2 had a growth-promoting effect on RCC cells *in vivo*, a xenograft tumor growth assay was performed in NOD/SCID mice. Subcutaneous tumor growth of A498 cells with FOXA2 shRNA-mediated stable knockdown or scrambled control was monitored. Tumor cells with FOXA2 knockdown grew significantly slower than those with the scramble control in the same mouse (Fig. 2*G*). Correspondingly, tumor volume and weight in FOXA2 knockdown mice were significantly reduced compared to those in control mice (Fig. 2, *H* and *I*). These data further support that FOXA2 promotes cell proliferation *in vitro* and RCC progression *in vivo*.

### FOXA2 directly activates the expression of HIF2α in RCC cells

**Figure 3:**
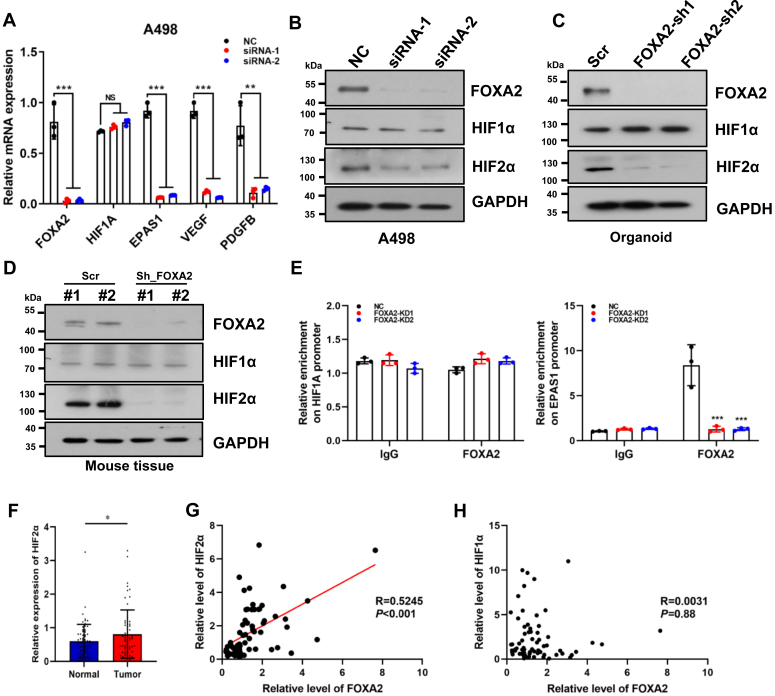
**Identification of HIF2α as a direct downstream target of FOXA2 in RCC.***A*, quantitative real-time PCR analysis of the effect of FOXA2 knockdown on the mRNA levels of HIF1A, EPAS1, VEGR and PDGFB relative to the NC control. The results are shown as the mean ± SD from three independent experiments. ∗∗*p* < 0.01, ∗∗∗*p* < 0.001. *B* and *C*, Western blot analysis of FOXA2, HIF1α and HIF2α in FOXA2-deficient A498 cells (*B*) and organoids (*C*). GAPDH was used as a loading control. The FOXA2 and GAPDH blots (*B*) were derived from Figure 2*A*. *D*, Western blot analysis of FOXA2, HIF1α and HIF2α in FOXA2-deficient A498 cell-derived xenograft tumor tissues. GAPDH was used as a loading control. *E*, FOXA2 was enriched at the promoter region of the EPAS1 gene but not at that of the HIF1A gene in A498 cells by ChIP analysis. Both promoters contain the consensus FOXA2 binding motif TAAACA. IgG was used as a negative control. The results are shown as the mean ± SD from three independent experiments. ∗∗*p* < 0.01. ∗∗∗*p* < 0.001. *NS*: no significance. *F*, HIF2α protein expression levels by quantitative analysis of the density of protein bands from Western blot in Fig. S2 relative to the normal tissues (n = 67). ∗*p* < 0.05. *G*, Pearson correlation scatter plot of H scores (protein levels) of FOXA2 and HIF2α protein levels in renal cancer tissues (n = 67). *H*, no correlation between FOXA2 and HIF1α protein levels in renal cancer tissues (n = 67).

Next, we investigated the potential mechanisms by which FOXA2 knockdown inhibited RCC progression. Since HIF1α and HIF2α have been reported to be critical for the progression of RCC (24, 25, 26), we knocked down FOXA2 in A498 cells and examined its effect on the expression of HIF1α and HIF2α. Interestingly, we found that knockdown of FOXA2 significantly reduced the EPAS1 (or HIF2A, encoding HIF2α) mRNA level compared to the NC control, while the HIF1A (encoding HIF1α) mRNA level remained unchanged according to quantitative RT-PCR analysis (Fig. 3*A*). We also observed that VEGF and PDGFB mRNA levels were significantly decreased in FOXA2 knockdown cells compared to NC control cells (Fig. 3*A*). Western blot analysis showed that the knockdown of FOXA2 significantly reduced the HIF2α protein level compared to the NC control, while the HIF1A protein level remained unaltered in A498 cells (Fig. 3*B*). Similar results were also obtained in RCC organoid cells and xenografted tumor tissues (Fig. 3, *C* and *D*). In fact, ChIP experiments demonstrated that FOXA2 could be significantly enriched at the EPAS1 promoter, while no enrichment was observed at the HIF1A promoter (Fig. 3*E*). Conversely, knocking down either HIF1A or EPAS1 did not affect FOXA2 expression, indicating that FOXA2 may act upstream of HIF2α (Fig. S4*A*). Notably, significantly higher expression levels of HIF2α were observed in renal tumor tissues than in matched adjacent normal tissues from RCC patients (Fig. 3*F*). In addition, the expression of HIF2α and FOXA2 positively correlated well across the RCC samples analyzed (Fig. 3*G*). In contrast, we found no correlation between HIF1α and FOXA2 protein levels in clinical RCC tissues (Figs. 3*H* and S4*B*). These results suggest that FOXA2 could directly activate HIF2α expression in RCC cells.

### HIF2α is involved in FOXA2-promoted RCC cell proliferation

**Figure 4:**
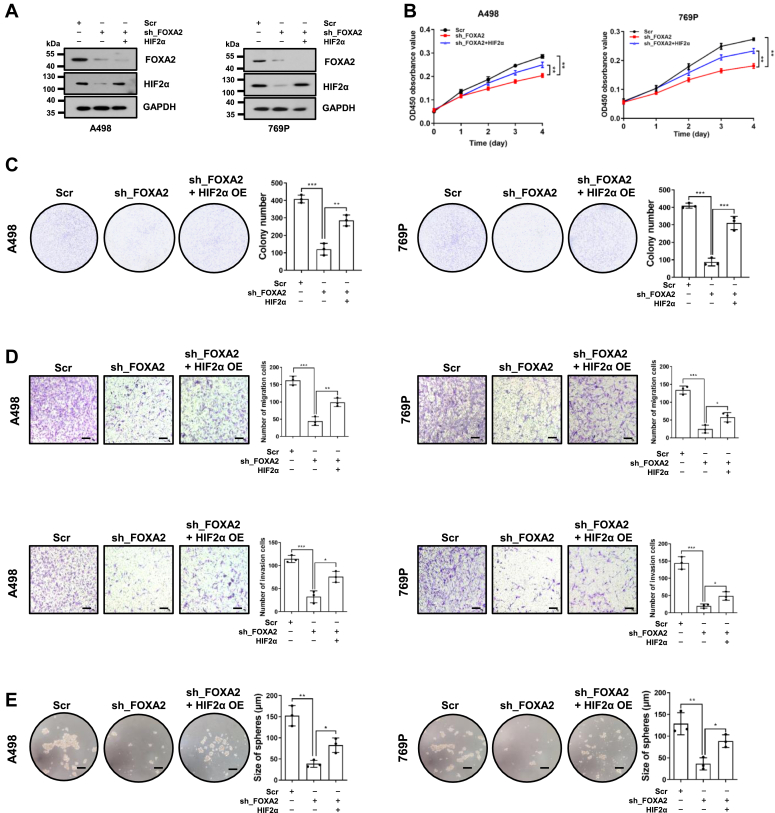
**FOXA2 promotes RCC cell proliferation by activating HIF2α.***A*, Western blotting analysis of the indicated proteins in A498 or 769P cells infected with scrambled control shRNA, FOXA2 shRNA or FOXA2 shRNA with the restoration of HIF2α expression. GAPDH was used as a loading control. *B*, expression of HIF2α reversed the cell growth inhibition mediated by FOXA2 shRNA in A498 or 769P cells, as determined by CCK-8 assays. The results are shown as the mean ± SD from three independent experiments. ∗∗*p* < 0.01. *C*, expression of HIF2α reversed the cell colony growth inhibition mediated by FOXA2 shRNA in A498 or 769P cells, as shown by colony formation assays. The results are shown as the mean ± SD from three independent experiments. ∗∗*p* < 0.01, ∗∗∗*p* < 0.001. *D*, expression of HIF2α reversed the cell migration inhibition (*top panels*) and cell invasion inhibition (*bottom panels*) mediated by FOXA2 shRNA in A498 or 769P cells, as shown by transwell assays. Scale bars, 100 μm. The results are shown as the mean ± SD from three independent experiments. ∗*p* < 0.05, ∗∗*p* < 0.01, ∗∗∗*p* < 0.001. *E*, expression of HIF2α reversed the cell tumorsphere growth inhibition mediated by FOXA2 shRNA in A498 or 769P cells, as determined by tumorsphere assays. Scale bars, 100 μm. The results are shown as the mean ± SD from three independent experiments. ∗*p* < 0.05, ∗∗*p* < 0.01, ∗∗∗*p* < 0.001.

Given that HIF2α is a key direct downstream target of FOXA2 in RCC cells, we examined whether HIF2α is critical for FOXA2-promoted RCC progression. We found that FOXA2 knockdown significantly reduced the cell growth rate, the numbers of cell colonies formed after culture, migratory and invasive capabilities, and stemness of A498 and 769P cells compared to Scr control cells (Fig. 4, *A*–*E*). Importantly, these phenotypes could be significantly reversed by enforced overexpression of HIF2α in FOXA2 knockout A498 and 769P cells (Fig. 4, *A*–*E*). These results further indicated that HIF2α plays a key role in FOXA2-promoted RCC progression.

### FOXA2 stability is regulated by VHL in RCC cells

**Figure 5:**
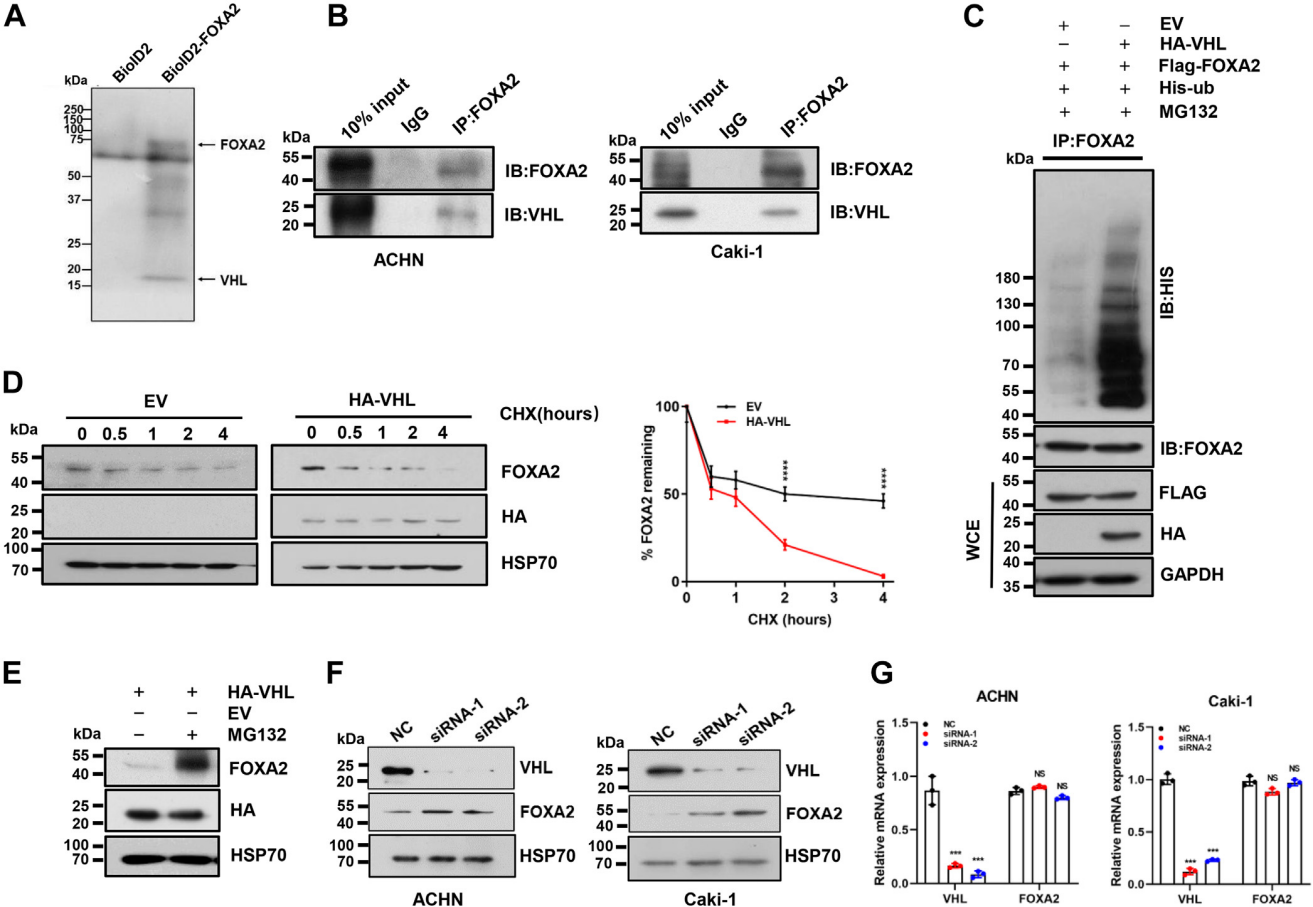
**FOXA2 stability is regulated by VHL.***A*, proteins biotinylated by BioID2-only or BioID2-FOXA2 in ACHN cells were pulled down by streptavidin agarose resins and analyzed by mass spectrometry. The bands corresponding to FOXA2 and VHL are shown. *B*, coimmunoprecipitation of endogenous FOXA2 and VHL from ACHN and Caki-1 cells. *C*, FOXA2 was ubiquitinated by VHL. HEK293T cells were transfected with the indicated plasmids in the presence of MG132 (5 μM) for 30 h. Cell lysates were immunoprecipitated with anti-FOXA2 antibody. The precipitates were detected using His and FOXA2 antibodies. Input cell lysates were detected using Flag and HA antibodies. GAPDH was used as a loading control. *D*, ACHN cells were transfected with empty vector (EV) or HA-VHL expression plasmid. After 36 h, the cells were treated with cycloheximide (CHX, 10 μg/mL) for the indicated times. The levels of FOXA2 and VHL were determined by immunoblotting, and GAPDH was used as a loading control (*left panel*). The amount of FOXA2 at each time point is plotted as a percentage of the amount at the start of the chase normalized to HSP70 and represents the means ± SDs from three independent experiments (*right panel*). ∗∗∗∗*p* < 0.0001. *E*, the decreased tendency of FOXA2 by VHL was rescued by MG132. The levels of FOXA2 and HA-VHL were analyzed by western blotting with the indicated antibodies. HSP70 was used as a loading control. *F*, the impact of VHL on FOXA2 protein abundance. ACHN and Caki-1 cells were transfected with VHL siRNAs or NC, and the levels of VHL and FOXA2 were determined by Western blotting with the indicated antibodies. HSP70 was used as a loading control. *G*, the mRNA levels of VHL and FOXA2 were analyzed by qRT‒PCR in ACHN and Caki-1 cells transfected with VHL siRNAs or NC. ∗∗*p* < 0.01.

Since high levels of FOXA2 expression correlate with poor prognosis in RCC and upregulated FOXA2 has the potential to promote RCC progression, we decided to probe how FOXA2 protein levels were regulated in RCC cells. Initially, we examined the FOXA2 protein levels in four RCC cell lines, A498, 769P, ACHN, and Caki-1 cells. We found that FOXA2 levels in A498 and 769P cells were significantly higher than those in ACHN and Caki-1 cells (Fig. S5*A*). Intriguingly, after reviewing the literature, we found that VHLs were mutated in A498 and 769P cells but normal in ACHN and Caki-1 RCC cells (27). Thus, we wondered whether FOXA2 expression levels were associated with VHL in these RCC cells, although we could not exclude the possibility that there might be transcriptional regulation involved. Nevertheless, we tested the use of a proximity-dependent biotin identification (BioID) approach to identify proximity interactions of FOXA2 in ACHN cells. In this system, FOXA2 was fused to BioID2 (28), a biotin ligase isolated from *Aquifex aeolicus*, and coexpressed in ACHN cells. An empty vector expressing BioID2 was used as a control. Cellular lysates were purified by streptavidin agarose resins, and elutes were analyzed by mass spectrometry to identify proteins that could interact with FOXA2. Among the interacting proteins identified, VHL was shown to be one of the most abundant proteins (Fig. 5*A*; Table S4). To confirm whether VHL could interact with FOXA2 in cells, we performed endogenous coimmunoprecipitation (Co-IP) experiments in ACHN and Caki-1 cells. As shown in Figure 5*B*, VHL was verified to interact with FOXA2 in both cell lines. In fact, FOXA2 could be ubiquitinated in the presence of VHL and migrated as a high molecular weight polyubiquitinated species in ACHN cells (Fig. 5*C*). Next, we examined the influence of VHL on FOXA2 protein stability by determining the half-life of endogenous FOXA2 in ACHN cells transfected with HA-tagged VHL or the empty vector as a control. Cellular extracts were prepared at various time points after cycloheximide treatment, subjected to SDS‒PAGE, and immunoblotted with antibodies against FOXA2 or HSP70 as a control. As shown in Figure 5*D*, the half-life of FOXA2 in ACHN cells transfected with HA-VHL was significantly reduced compared to the vector control, suggesting that the stability of FOXA2 was dependent on VHL-mediated degradation. In fact, MG132, a proteasome inhibitor, was shown to significantly protect FOXA2 protein from degradation, further validating the effect of VHL on FOXA2 stability (Fig. 5*E*).

To further test whether VHL functioned as an E3 ubiquitin ligase against FOXA2 in ACHN and Caki-1 cells, we knocked down VHL in these cells and examined the FOXA2 expression levels by Western blot and quantitative RT‒qPCR analysis. We found that the protein levels of FOXA2 were significantly increased in VHL knockdown cells compared to NC control cells (Fig. 5*F*). We noted that the mRNA levels of FOXA2 were unchanged between VHL knockdown cells and NC control cells (Fig. 5*G*). To examine the effect of FOXA2 on ACHN and Caki-1 cells, we overexpressed FOXA2 in these cells. We showed that overexpression of FOXA2 significantly increased the cell growth rate, the numbers of RCC cell colonies formed after culture, migratory and invasive capability, and the tumorsphere-formation activity of two cell lines compared to vector-transfected cells (Fig. S5, *B*–*F*). Together, these results suggest that FOXA2 stability is posttranscriptionally regulated by VHL in RCC cells.

### K264 is the major ubiquitination residue by VHL in FOXA2 in RCC cells

**Figure 6:**
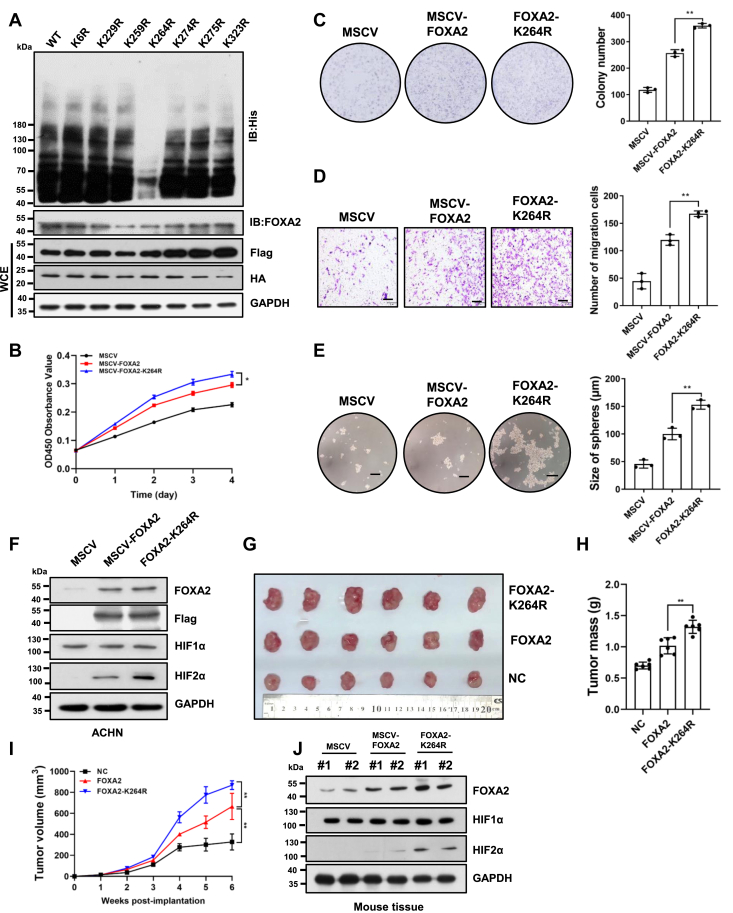
**K264 is the major ubiquitinated site in FOXA2.***A*, HEK293T cells were transfected with Flag-tagged wild-type FOXA2 (WT) or its mutants bearing Lys to Arg substitutions (K6R, K229R, K264R, K274R, K275R, and K323R) as in Figure 5*C*. Cell lysates were immunoprecipitated with anti-Flag M2 beads. The precipitates were detected with His and FOXA2 antibodies. Whole-cell lysates were analyzed by immunoblotting with Flag and HA antibodies. GAPDH was used as a loading control. *B*, cell growth was determined by CCK-8 assays in ACHN cells transfected with MSCV-FOXA2 (WT), FOXA2-K264R (mutant), or MSCV. The results are shown as the mean ± SD from three independent experiments. ∗*p* < 0.05. *C*, colony formation assay of ACHN cells overexpressing FOXA2, FOXA2-K264R, or vector alone (MSCV). The number of colonies formed by the indicated cells was quantified. The results are shown as the mean ± SD from three independent experiments. ∗∗∗*p* < 0.001. *D*, transwell assay of ACHN cells overexpressing FOXA2, the FOXA2-K264R mutant or MSCV. The number of migrating cells was quantified. The results are shown as the mean ± SD from three independent experiments. Scale bars, 100 μm. ∗∗*p* < 0.01. *E*, tumor sphere formation assay of ACHN cells overexpressing FOXA2, the FOXA2-K264R mutant or MSCV. Scale bars, 100 μm. The size of the spheres formed by the indicated cells was quantified. The results are shown as the mean ± SD from three independent experiments. ∗∗*p* < 0.01. *F*, ACHN cells were transfected with MSCV-FOXA2, FOXA2-K264R plasmids, or MSCV as a negative control. The levels of FOXA2, Flag, HIF1α, and HIF2α were analyzed by western blotting. GAPDH was used as a loading control. *G*–*I*, Photograph (*G*), tumor masses (*H*) or tumor volumes (*I*) of negative control (NC) MSCV-, FOXA2-or FOXA2-K264R-transfected ACHN cell-derived xenograft tumors at day 35. The results are shown as the mean ± SD (n = 6). ∗∗*p* < 0.01. *J*, Western blot analysis of FOXA2, HIF1α, and HIF2α in MSCV-, FOXA2-or FOXA2-K264R-transfected ACHN cell-derived xenograft tumors. GAPDH was used as a loading control.

After confirming VHL-mediated ubiquitination of FOXA2, we determined which residues in FOXA2 could be targeted by VHL. We initially searched for potential ubiquitin-conjugation sites on FOXA2 by using Ubisite (29). By ranking the scores of each site provided by the software, we listed seven residues (K6, K229, K259, K264, K274, K275, and K323) that could be candidate sites for ubiquitination by VHL (Table S5). To determine the major residues for VHL ubiquitination, we constructed corresponding FOXA2 mutants harboring a single Lys-to-Arg (K-R) substitution and performed *in vivo* ubiquitination assays. We observed that ubiquitination of the K264R mutant of FOXA2 was significantly reduced compared to that of the wild type, while ubiquitination of other mutants remained similar to that of the wild type (Fig. 6*A*). To evaluate the effect of the FOXA2-K264R mutant on cell proliferation, we transfected ACHN cells with plasmids overexpressing wild-type FOXA2 or the FOXA2-K264R mutant. We found that the FOXA2-K264R mutant significantly increased the cell growth rate in ACHN cells compared to wild-type FOXA2 (Fig. 6*B*). In addition, the FOXA2-K264R mutant significantly increased the number of cell colonies formed after culture and the migratory and invasive capability of RCC cells compared to wild-type FOXA2, as demonstrated by colony formation and transwell assays (Fig. 6, *C* and *D*). Furthermore, we observed that tumorsphere-formation activity was also significantly elevated in cells harboring FOXA2-K264R compared to cells harboring wild-type FOXA2 (Fig. 6*E*). Consistent with these results, Western blot analysis showed that HIF2α expression in FOXA2-K264R mutant cells was significantly higher than that in FOXA2 wild-type cells (Fig. 6*F*). Once again, HIF1α expression remained unchanged between FOXA2-K264R mutant and wild-type cells (Fig. 6*F*). To examine whether the FOXA2-K264R mutant had a growth-accelerating effect on RCC cells *in vivo*, a xenograft tumor growth assay was performed in NOD/SCID mice. Subcutaneous tumor growth of ACHN cells with the FOXA2-K264R mutant or wild-type FOXA2 was monitored. Tumor cells with the FOXA2-K264R mutant grew significantly faster than those with wild-type FOXA2 in the same mouse (Fig. 6*G*). Correspondingly, tumor volume and weight in FOXA2-K264R mutant mice were significantly increased compared to those in wild-type FOXA2 mice (Fig. 6, *H* and *I*). Western blot analysis showed that HIF2α expression in FOXA2-K264R mutant tissues was significantly higher than that in FOXA2 wild-type tissues (Fig. 6*J*). These results indicated that K264 was the major residue in FOXA2 ubiquitinated by VHL in RCC cells and that the FOXA2-K264R mutant resisting ubiquitination accelerated RCC progression.

## Discussion

**Figure 7:**
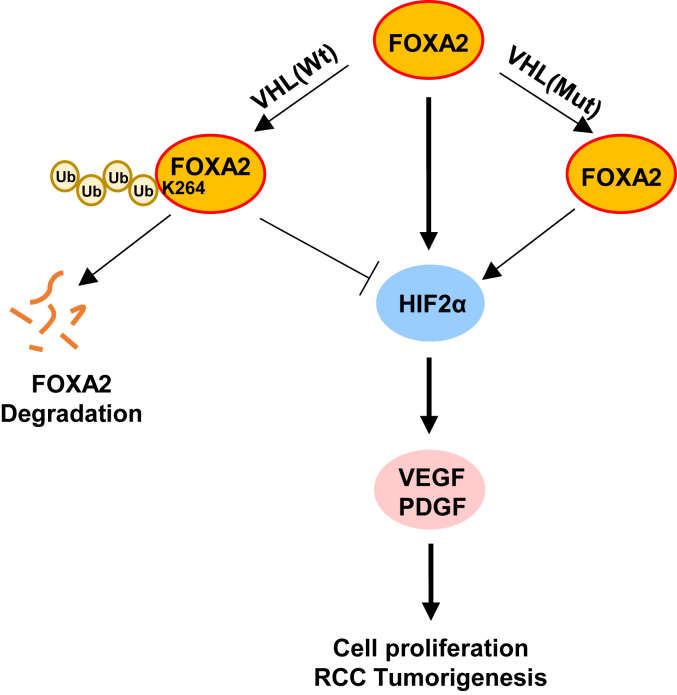
**A hypothetical model of the FOXA2-HIF2α axisregulating cell proliferation and RCC progression.**

RCC is a highly aggressive tumor with poor patient outcomes (2). A large body of evidence has shown that aberrant regulation of the HIF2 signaling pathway is tightly linked to RCC (24, 30). In the present study, we found that FOXA2 functions as an upstream transcription activator of HIF2α to promote RCC progression (Fig. 7). Intriguingly, we found that FOXA2 could also be targeted by VHL, which regulates its protein stability in RCC cells although we cannot exclude the possibility that FOXA2 could also be transcriptionally regulated by an unknown mechanism that plays a role in RCC (Fig. 7). These results may help update our knowledge about the regulatory mechanism of HIF2α in RCC progression, thus providing an alternative therapeutic strategy for RCC tumors.

Since FOX family transcription factors play pivotal roles in organogenesis during development and in the maintenance of physiological homeostasis, alterations of FOX family genes are implicated in carcinogenesis through gene amplification, chromosomal translocation, and transcriptional regulation (12, 13, 14). Studies have shown that FOXA2 could function as a regulator in lung cancer, breast cancer, prostate cancer, bladder cancer, and Barrett’s metaplasia (15, 16, 17, 18, 31). In this study, we found that FOXA2 is directly bound to the promoter region of EPAS1, activating its expression. However, we cannot exclude the possibility that other pathways or downstream targets could also be involved in promoting RCC progression by FOXA2. The FOXA2 proteins, as pioneer factors, not only establish the competence of the ventral foregut endoderm but also facilitate the binding of nuclear hormone receptors to their targets in multiple organ systems in adult mammal (12). Therefore, it would be interesting to test whether FOXA2 could collaborate with the glucocorticoid receptor (GR) to regulate HIF2α and other downstream target genes under pathological conditions (12).

HIF2α has an important role in the pathogenesis of ccRCC. VHL gene mutation is a common phenomenon in clear cell renal cell carcinoma, accounting for 80 to 90% of patients with RCC (2). Studies have shown that the mutation of VHL could protect its substrate HIF2α from degradation by ubiquitination (32), leading to the activation of its downstream signaling pathways, such as VEGF and PDGF, to promote angiogenesis, proliferation and tumorigenesis (24). Interestingly, in our study, we found that FOXA2 could also be targeted by VHL. Mutation of K264 in FOXA2 resisted ubiquitin-mediated degradation and increased the proliferative cell capability of RCC *in vitro* and *in vivo*. In line with these results, we showed that FOXA2 expression in ACHN or Caki-1 cells (wild-type VHL gene) was indeed significantly lower than that in A498 and 769P cells (mutant VHL gene) (27). Intriguingly, when FOXA2 was overexpressed in ACHN or Caki-1 cells, we found that FOXA2 could promote the proliferation and stemness of RCC cells. These results agree with previous experiments in which reactivation of HIF2α can override VHL’s ability to suppress xenograft formation by ccRCC cell lines lacking *VHL* in preclinical models (33). Alternatively, there is potential competition between FOXA2 and HIF-2α for VHL in RCC cells. More FOXA2 may occupy VHL and prevent oxygen-dependent HIF-2α ubiquitination and degradation, which in turn results in RCC tumorigenesis. Thus, we favor that FOXA2 functions as an oncogene in RCC development regardless of the VHL mutation in this context.

In conclusion, this study revealed a novel role of FOXA2 in RCC development. We showed that FOXA2 activates HIF2α expression to promote renal cell carcinoma progression and is regulated by VHL. Previously, many studies have focused on the development of HIF2α-targeted therapies for patients, and the FDA approved a HIF2α inhibitor, belzutifan (Welireg, Merck), for adult patients with VHL-associated tumors in 2021 (30). However, the challenge of adverse side effects and drug resistance recalls a better therapeutic strategy for RCC treatment (34). Our results suggest that FOXA2 can act as a key oncogene in RCC and may represent an alternative novel therapeutic target for RCC patients.

## Experimental procedures

### Tissue samples, tissue microarray and immunohistochemical staining

Fresh human tissues (paired RCC and adjacent normal tissues) were collected from 67 patients undergoing surgery at Jiangsu Province Hospital, Nanjing, China. Collection and analysis of the human tissue were approved by the ethics committee of Jiangsu Province Hospital and conformed to the tenets of the Declaration of Helsinki, and all patients provided informed consent. TMA chips containing a total of 75 pairs of ccRCC samples and matched normal samples with clinical pathological data were obtained from Shanghai Biochip Co, Ltd. IHC staining was performed by Nanjing Microworld Biotechnology Co, Ltd. The tissue slides were incubated with primary antibodies specific for FOXA2 followed by incubation with horseradish peroxidase-conjugated secondary antibody. IHC staining of FOXA2 in the TMA was scored independently by two experienced pathologists blinded to the clinical data. The staining of cells was analyzed according to staining intensity on a scale of 0 to 3 (0 = *negative*, *weak* = 1, *moderate* = 2, *strong* = 3). The H score was calculated by multiplying the scale score times the percent of cells having that score and then summing the products across all scale scores, that is, H score (0–300 scale) = 3 × (% at 3) + 2 × (% at 2) + 1 × (% at 1). The clinical features of the patients are listed in Table 1. For survival analyses, patient survival was stratified by expression of the gene of interest between two groups: high (≥ median value) and low (< median value), and is presented as Kaplan–Meier plots and tested for significance using log-rank tests. Degrees of correlation between stained proteins for determining patient protein expression patterns were assessed *via* Pearson correlation analysis.

### Cell lines and cell culture

The human renal cell carcinoma cell lines A498, 769P, ACHN, and Caki-1 were purchased from the Cell Bank of the Chinese Academy of Sciences and cultured in several media according to ATCC handling information. All cell lines were supplemented with 10% fetal bovine serum and 1% (v/v) penicillin/streptomycin. Cells were cultured in a humidified atmosphere of 5% CO_2_ at 37 °C. All cell lines were confirmed to be mycoplasma-free and authenticated by Genetic Testing Biotechnology Corporation using short tandem repeat (STR) profiling.

### RNA interference, plasmid, and infection

siRNAs against FOXA2 and VHL were synthesized by KeyGEN BioTECH CO, Ltd. A final concentration of 50 nM siRNA was used to transiently transfect cells with Lipofectamine 3000. The siRNA sequences targeting FOXA2 were as follows:

FOXA2-siRNA-1: 5′-AAAUGGACCUCAAGGCCUA-3′

FOXA2-siRNA-2: 5′-GAACACCACUACGCCUUCA-3′

The siRNA sequences targeting VHL were as follows:

VHL-siRNA-1: 5′-GAACAUGUCGUCGUACGUG-3′

VHL-siRNA-2: 5′-GCAGAUACCUCCUACUACC-3′

shRNA target sequences for interference of FOXA2 were inserted into pLKO.1. The oligonucleotides were as follows:

FOXA2 shRNA-1:

5′-CCGGGAACGGCATGAACACGTACATCTCGAGATGTACGTGTTCATGCCGTTCTTTTTG-3′

FOXA2 shRNA-2:

5′-CCGGCTACGCCAACATGAACTCCATCTCGAGATGGAGTTCATGTTGGCGTAGTTTTTG-3′

A scramble sequence was used as a negative control (NC).

shRNA lentivirus was produced by cotransfection of pLKO.1 and the packaging plasmids pMD2G and psPAX2 in HEK293T cells. The viral supernatant was collected at 48 h after transfection. The renal cell carcinoma cells were incubated with supernatants supplied by Polybrene. The cells expressing FOXA2-shRNA were selected with puromycin.

For overexpression of FOXA2, HIF2α, and VHL, human FOXA2, HIF2α, and VHL cDNA were cloned into the retroviral vector plasmid MSCV. Constructed plasmids were transiently transfected into cells by Lipofectamine 3000.

Site-directed mutagenesis was performed using the Mut express II mutagenesis Kit according to the manufacturer’s protocols. The sequences of the primers are listed in Table S1.

### RNA isolation, quantitative real-time PCR (qRT‒PCR), and ChIP assay

Total RNA from tissues and cells was extracted with TRIzol reagent according to the manufacturer’s instructions. Complementary DNAs (cDNAs) were synthesized using HiScrip III RT SuperMix. qRT‒PCR analysis was performed using AceQ qPCR SYBR Green Master Mix (Vazyme) according to the manufacturer’s protocols. The relative mRNA expression of each gene was normalized to the expression of the loading control gene GAPDH. The sequences of the primers are listed in Table S2.

ChIP assays were performed as described previously (35). Normal rabbit IgG served as the control. The primer sequences for ChIP are listed in Table S3.

### Western blot analysis and immunoprecipitation

Protein from tissues and cells was extracted by RIPA lysis buffer and cell lysis buffer for western blots, respectively. Equal amounts of proteins were separated by SDS‒PAGE and then transferred to polyvinylidene difluoride (PVDF) membranes by semidry blotting. The membranes were blocked at room temperature in 5% (w/v) nonfat milk diluted with PBST for 1 h and incubated overnight with primary antibodies at 4 °C. Primary antibodies against FOXA2 (Abcam, Cat# ab256493, 1:1000), HIF1α (Abcam, Cat# ab179483, 1:1000), HIF2α (Abcam, Cat# ab207607, 1:1000), VHL (Abcam, Cat# ab270968, 1:1000), HA-tag (Proteintech, Cat# 51064-2-AP, 1:10000), Flag-tag (Proteintech, Cat# 66008-4-Ig, 1:100000), His-tag (Proteintech, Cat# 66005-1-Ig, 1:100000), Hsp70 (Proteintech, Cat# 10995-1-AP, 1:100000) and GAPDH (Proteintech, Cat# 10494-1-AP, 1:200000) were used. HRP-conjugated Affinipure goat anti-rabbit IgG HA-tag (Proteintech, Cat# SA00001-2, 1:10000) or goat anti-mouse IgG (Proteintech, Cat# SA00001-1, 1:10000) were used as the secondary antibody. After incubation with secondary antibodies at room temperature for 1 h, the blots were visualized using an ECL detection system. The blots were exposed to X-ray film and then scanned by an Epson Perfection V700 Photo Scanner. Densitometry of Western blot was analyzed by ImageJ software.

Flag-FOXA2 or HA-VHL was expressed in ACHN and Caki-1 cells seeded in 10-cm dishes. Cells were collected and lysed in cell lysis buffer containing 1x proteinase inhibitor for western blotting. After spinning down, the supernatants were incubated with anti-Flag M2 beads or protein A/G beads preincubated with anti-HA antibody at 4 °C for 2 h. The beads were then washed three times with NETN (250 mM NaCl, 5 mM EDTA, 50 mM Tris-HCl, 0.5% NP-40) and eluted with PBS. The proteins were then prepared and separated on 12% SDS‒PAGE gels and subjected to western blotting. For endogenous protein, ACHN and Caki-1 cells were seeded in three 10-cm dishes and lysed, and supernatants were collected by centrifugation. After incubation with anti-FOXA2 antibody or rabbit IgG for 4 h, cell extracts were then incubated with protein A/G beads for 2 h. The precipitates were washed with NETN buffer three times and then subjected to standard immunoblotting.

### CCK8 and colony formation assays

Cell viability was examined using the Cell Counting Kit-8 following the manufacturer’s protocol, and the absorbance at 450 nm was assessed by a multifunction microplate reader (Safire, TECAN). For the colony formation assay, cells were seeded in 6-well plates at an equal density of 1000 cells per well, and the culture medium was refreshed every 4 days for 2 weeks. Colonies were fixed with methanol and then stained with crystal violet. After washing with distilled water, the colonies were counted and photographed.

### Migration, invasion, and tumorsphere assays

For the cell migration assay, cells were seeded into the upper chamber in serum-free medium at an equal density of 3 × 10^4^ cells per chamber, and the bottom chamber was supplemented with medium containing 15% FBS. After 20 h, the cells that passed through the 8-μm pore-size polycarbonate filter and migrated to the lower membrane surface were fixed with methanol and then stained with crystal violet. After washing with distilled water, the cells were counted and photographed under a microscope at 100× magnification. The invasion assay was performed as summarized in the cell migration assay, except that the upper chamber was coated with Matrigel. For the tumor sphere assay, the cells were seeded in 6-well plates with an ultralow attachment surface (Corning, Roche) at an equal density of 5000 cells per well with 4 ml DMEM/F12 culture medium supplemented with B27, basic fibroblast growth factor (bFGF), and epidermal growth factor (EGF). After incubation in a humidified atmosphere of 5% CO2 at 37 °C for 10 to 14 days, the tumor spheres were assessed and photographed.

### BioID2 system and mass spectrometry

To identify proteins interacting with FOXA2, BioID2 or BioID2-FOXA2 vector was transfected into ACHN cells seeded in 10-cm dishes. Twenty-four hours after transfection, the culture medium was replaced with a medium containing 50 μM biotin for 18 h. Cells were then harvested and lysed in lysis buffer (50 mM Tris-HCl pH 7.4, 500 mM NaCl, 0.4% SDS [w/v]). After sonication, the supernatants were collected and incubated with streptavidin agarose resins. The beads were then washed with wash buffer (8 M urea, 50 mM Tris-HCl pH 7.4) and used in western blotting analysis or subjected to LC-MS/MS analysis (Data are available *via* ProteomeXchange with identifier PXD043802).

### Cycloheximide chase assay and ubiquitination assay

The indicated constructs were transfected into HEK293T cells and grown for 24 h. Cycloheximide (CHX) was then added to suppress newborn protein synthesis. Equal amounts of cells were harvested at the indicated time points. The protein level of FOXA2 in different groups was detected by immunoblotting.

To verify the ubiquitination of FOXA2 *in vivo*, Flag-tagged FOXA2 was immunoprecipitated from cells treated with the proteasome inhibitor MG132. Plasmid pCMV-His-UB (#P4836) was obtained from Miaoling Biology. Cells were collected and lysed in cell lysis buffer for western blotting. After centrifugation, the supernatants were subjected to western blotting to determine the expression of FOXA2 and VHL or incubated with anti-Flag M2 beads overnight at 4 °C. The beads were washed three times and subjected to immunoblotting using anti-His antibody.

### Organoids

The RCC patient-derived organoid protocol was described previously (36). Briefly, fresh tissue was cut into small pieces <1 mm^3^ in size with a scalpel, and then the pieces were washed with PBS and digested with collagenase P. After digestion, the sample was filtered through a 70 μm cell strainer followed by a 40 μm cell strainer. Next, the cell suspension was resuspended in MACS buffer and sorted by anti-CD45 microbeads following RBC erythrocyte lysis buffer incubation. The cells were then suspended in a prechilled growth medium containing DMEM-F12, B-27, EGF, FGFb, heparin, penicillin‒streptomycin, and amphotericin B. After mixing with Matrigel, the mixture was plated on a 48-well plate, and an organoid growth medium was added. The organoids were incubated at 37 °C in a 5% CO_2_ incubator. The medium was refreshed every 3 days, and the organoid could be passaged every 1 to 2 weeks.

### *In vivo* xenograft growth assay

Animal experiments were performed in accordance with the National Institutes of Health Guide for the Care and Use of Laboratory Animals and were approved by the Institutional Review Board of Nanjing University (Nanjing, China). The sample size was chosen with adequate power on the basis of the literature and our previous experience (37), and for each experiment, it is indicated in the figure legend. Prior to carrying out the experiment, mice were randomly assigned to different treatment groups. Four-week-old nonobese diabetic/severe combined immunodeficiency (NOD/SCID) female mice were purchased from the Model Animal Research Center of Nanjing University (Nanjing, China). After adapting to the environment for 2 weeks, infected A498 cells were suspended in MEM medium without serum containing 10% Matrigel and injected subcutaneously (s.c.) into the right flank of each mouse (5 × 10^6^ cells per mouse). Tumor size was measured with a digital caliper every week, and tumor volume was calculated using the formula: Volume (cm^3^) = 0.5 × (length × width^2^). Five weeks later, the mice were sacrificed, and the tumor burden was examined, weighed, and photographed.

### Statistical analysis

Data analysis was performed with the statistical program GraphPad Prism (GraphPad Prism). The results are presented as the mean ± SD unless otherwise indicated. Statistical analyses were performed using two-tailed Student’s *t* test to derive the significance of the differences between the two groups. *p* < 0.05 was considered to be significant.

## Data availability

The LC‒MS/MS analysis data are available *via* ProteomeXchange with identifier PXD043802. The source data of this study are available from the corresponding auther upon reasonable request.

## Supporting information

This article contains supporting information.

## Conflict of interest

The authors declare that they have no conflicts of interest.
