# Supplementary material for: FOXA2 activates HIF2α expression to promote tumor progression and is regulated by the E3 ubiquitin ligase VHL in renal cell carcinoma
Source: J Biol Chem. 2023 Dec 10;300(1):105535. doi: 10.1016/j.jbc.2023.105535 (PMC10801253; doi:10.1016/j.jbc.2023.105535)
Supplement: Supporting Tables S1–S5 and Figures S1–S5 [file mmc1.docx]

**FOXA2 activates HIF2α expression to promote tumor progression and is regulated by the E3 ubiquitin ligase VHL in renal cell carcinoma**

Dongjun Yang^1^, Qixiang Li^1^, Peifen Lu^1^, Dongliang Wu^1^, Wenyang Li^1^, Xingjun Meng^1^, Mengying Xing^1^, Wenbing Shangguan^1^, Bing Chen^1^, Jie Yang^2^, Zhihong Zhang^2^, Zengjun Wang^2^, David C. S. Huang^3^, and Quan Zhao^1,*^

^1^The State Key Laboratory of Pharmaceutical Biotechnology, Department of Hematology, the Affiliated Drum Tower Hospital of Nanjing University Medical School, China-Australia Institute of Translational Medicine, School of Life Sciences, Nanjing University, Nanjing, China; ^2^Department of Urology and Pathology, The First Affiliated Hospital of Nanjing Medical University, Nanjing, China. ^3^Department of Medical Biology, The Walter and Eliza Hall Institute of Medical Research, University of Melbourne, Melbourne, VIC, Australia

**Supplementary information**

**Supplementary Figure S1. A,** Immunohistochemical (IHC) staining of FOXA2 in clear cell renal cell carcinoma tissues of patients (Tnmor, n = 75) or adjacent normal tissues (Normal, n = 75). **B,** Transcript levels of FOXA2 in normal (n=100) and RCC tumor samples (n=93) from ccRCC data obtained from GEPIA databases. ***P<0.01.*

**Supplementary Figure S2.** FOXA2 and HIF2α expression in RCC tissues and adjacent normal tissues. Western blot analysis of FOXA2 and HIF2α in 67 pairs of RCC tissues (**T**) and adjacent normal tissues (**N**). GAPDH was used as a loading control.

**Supplementary Figure S3.** FOXA2 promotes cell proliferation in RCC organoids. **A,** Representative images of RCC organoids transfected with Scr shRNA or shRNA against FOXA2 at day 4. Scale bar, 100 μm. **B,** The size of the organoid formed in (**a**) was calculated. The results are shown as the mean ± SD from three independent experiments. ****P<0.001.* **C,** The effect of FOXA2 knockdown on the growth of RCC organoids. The results are shown as the mean ± SD from three independent experiments. ****P<0.001*. **D,** Representative images of RCC tissue- and adjacent normal tissue-derived organoids at day 7. Scale bar, 100 μm. **E,** Growth plot of RCC tissue- and adjacent normal tissue-derived organoids from day 1 to day 7. The results are shown as the mean ± SD from three independent experiments. ***P<0.01, ***P<0.001*.

**Supplementary Figure S4. A,** No effect of HIF on FOXA2 expression. Western blot analysis of negative control (NC) siRNA-, HIF1A-siRNA- or EPAS1-siRNA-transfected ACHN cells with the indicated antibodies. GAPDH was used as a loading control. Representative results are shown from three independent experiments. **B,** HIF1α expression in RCC tissues and adjacent normal tissues. Western blot analysis of HIF1α in 67 pairs of RCC tissues (**T**) and adjacent normal tissues (**N**). GAPDH was used as a loading control.

**Supplementary Figure S5.** Overexpression of FOXA2 promotes proliferation of ACHN and Caki-1 cells. **A,** Western blot analysis of FOXA2 in ACHN, A498, Caki-1 and 769P cells. GAPDH was used as a loading control. **B,** Western blot analysis of FOXA2 overexpression in ACHN or Caki-1 cells. GAPDH was used as a loading control. (top panels). Quantitative real-time PCR analysis of FOXA2 mRNA levels in ACHN or Caki-1 cells transfected with MSCV or MSCV-FOXA2 (bottom panels). **C,** Cell growth assay of ACHN or Caki-1 cells transfected with MSCV-FOXA2 or MSCV. The results are shown as the mean ± SD from three independent experiments. ***P<0.01*. **D,** Colony formation assay of ACHN or Caki-1 cells transfected with MSCV-FOXA2 or MSCV. The number of colonies formed by the indicated cells was quantified. The results are shown as the mean ± SD from three independent experiments. ***P<0.01*. **E,** Transwell assay of ACHN or Caki-1 cells transfected with MSCV-FOXA2 or MSCV. Scale bars, 50 μm. The number of migrated cells was quantified. The results are shown as the mean ± SD from three independent experiments. ***P<0.01, ***P<0.001*. **F,** Tumor sphere formation assay of ACHN or Caki-1 cells transfected with MSCV-FOXA2 or MSCV. Scale bars, 100 μm. The size of the spheres formed by the indicated cells was quantified. The results are shown as the mean ± SD from three independent experiments. ****P<0.001*.

**Supplementary Table S1. Primers for FOXA2 site-directed mutagenesis**

| **Mutants** | **Direction** | **Sequence (5’-3’)** |
| --- | --- | --- |
| K6R | Forward | GCGGTGAGGATGGAAGGGCACGAG |
|  | Reverse | TTCCATCCTCACCGCTCCCAGC |
| K229R | Forward | CAGGGGCTCCTTCTGGACCCTGCACCCTGACT |
|  | Reverse | TCCAGAAGGAGCCCCTGCCGGGCTTGTCGGGCG |
| K259R | Forward | TGCGAGAGGCAGCTGGCGCT |
|  | Reverse | CAGCTGCCTCTCGCACTTGAAGCGCT |
| K264R | Forward | GCGCTGAGGGAGGCCGCAG |
|  | Reverse | GGCCTCCCTCAGCGCCAGCT |
| K274R | Forward | AGCGGCAGGAAGGCGGCC |
|  | Reverse | CGCCTTCCTGCCGCTGCCG |
| K275R | Forward | GGCAAGAGGGCGGCCGCC |
|  | Reverse | GGCCGCCCTCTTGCCGCTG |
| K323R | Forward | CCTGGGAGGGGGGACGCCGGCTGCGGCGCTGA |
|  | Reverse | GCGTCCCCCCTCCCAGGCCCCCTCGCTTGTGC |

**Supplementary Table S2. Primers for qRT-PCR**

| **Gene symbol** | **Direction** | **Sequence (5’-3’)** |
| --- | --- | --- |
| FOXA2 | Forward | GGAGCAGCTACTATGCAGAGC |
|  | Reverse | CGTGTTCATGCCGTTCATCC |
| GAPDH | Forward | GAAGGTGAAGGTCGGAG |
|  | Reverse | GAAGATGGTGATGGGATTTC |
| HIF1A | Forward | CACCACAGGACAGTACAGGAT |
|  | Reverse | CGTGCTGAATAATACCACTCACA |
| EPAS1 | Forward | CGGAGGTGTTCTATGAGCTGG |
|  | Reverse | AGCTTGTGTGTTCGCAGGAA |
| PDGFB | Forward | CTCGATCCGCTCCTTTGATGA |
|  | Reverse | CGTTGGTGCGGTCTATGAG |
| VEGF | Forward | AGGGCAGAATCATCACGAAGT |
|  | Reverse | AGGGTCTCGATTGGATGGCA |
| VHL | Forward | GCAGGCGTCGAAGAGTACG |
|  | Reverse | CGGACTGCGATTGCAGAAGA |

**Supplementary Table S3. Primers for ChIP analysis**

| **Fragments** | **Direction** | **Sequence (5’-3’)** |
| --- | --- | --- |
| HIF1A | Forward | AAGGCGCAGAGTCCC |
|  | Reverse | CTGAGGCACAGCTGGG |
| EPAS1 | Forward | GGAGTCCCACCCCGCTAC |
|  | Reverse | CTGCCCTGACCCTGGC |

**Supplementaty Table S4. Mass spectrometry results (unique peptides)**
